# Implementing prescriber-pharmacist collaboration to improve evidence-based medication prescribing using asynchronous, non-interruptive electronic health record notifications

**DOI:** 10.1186/s13012-025-01478-9

**Published:** 2025-12-15

**Authors:** Geoffrey D. Barnes, Seo Youn Choi, Michael SM Lanham, Michael P Dorsch, Joshua Errickson, Morris Fabbri, Anish Saraswat, F Jacob Seagull, Shawna N Smith

**Affiliations:** 1 Frankel Cardiovascular Center and Institute for Healthcare Policy and Innovation, University of Michigan, Ann Arbor, MI USA; 2 School of Public Health, University of Michigan, Ann Arbor, MI USA; 3 Department of Learning Health Sciences, Department of Obstetrics and Gynecology, University of Michigan, Ann Arbor, MI USA; 4 College of Pharmacy, Frankel Cardiovascular Center and Institute for Healthcare Policy and Innovation, University of Michigan, Ann Arbor, MI USA; 5 Department of Statistics, Consulting for Statistics, Computing & Analytics Research, University of Michigan, Ann Arbor, MI USA; 6 Center for History, Humanities, Arts, Social Sciences and Ethics in Medicine, University of Michigan, Ann Arbor, MI USA; 7 University of Michigan Medical School, Ann Arbor, MI USA; 8 School of Public Health and Institute for Healthcare Policy and Innovation, University of Michigan, Ann Arbor, MI USA

**Keywords:** Anticoagulants, Clinical decision support system, Pharmacists, Implementation science, Atrial fibrillation, Venous thromboembolism

## Abstract

**Background:**

Inappropriate prescribing of Direct Oral Anticoagulants (DOACs) is a leading cause of adverse outcomes. Electronic health record (EHR)-based notification strategies may support evidence-based prescribing and reduce adverse events. Engaging clinical pharmacists (vs. prescribers) through EHR-based notifications that review inappropriate DOAC prescribing may be an effective strategy for ensuring evidence-based medication prescribing.

**Methods:**

We conducted a pragmatic, single-center, parallel-group, randomized implementation trial using notifications (asynchronous EHR-based notifications) to prompt correction of inappropriate DOAC prescriptions that had arisen after the initial prescription of the DOAC (e.g., due to changes in patient condition). Notifications were sent for adult ambulatory patients with DOAC prescriptions not adhering to the package insert instructions or having significant drug-drug interactions. Notifications directed either to the prescribing clinician or to the clinical anticoagulation pharmacist, randomized at the prescriber level. The primary outcome was the proportion of notifications adopting any prescription change within 7 days. Moderator analyses examined the influence of prescriber, patient, and prescription characteristics.

**Results:**

From May 2023 to December 2024, 388 notifications for potentially inappropriate DOAC prescriptions among 183 prescribers were analyzed. Overall, 23.2% of notifications led to a prescription change within 7 days, 26% among prescriber-directed and 21% among pharmacist-directed notifications (p = 0.36). Nearly all (97.8%) changes made were clinically appropriate changes aligned with notification recommendations. Subgroup and moderator analyses showed that pharmacists made more changes than prescribers when errors were further from dosing cutoffs and managed cases with polypharmacy or complex thresholds more consistently. Clinical pharmacists spent an average of 7.9 min per notification.

**Conclusions:**

Prescribers and clinical pharmacists both responded similarly and consistently to correct inappropriate DOAC prescriptions in response to EHR asynchronous notifications. While pharmacists did not outperform prescribers overall, they demonstrated more nuanced application of medication prescribing guidelines in complex cases. Engaging clinical pharmacists directly may be an efficient implementation strategy for addressing medication prescribing issues. Optimal EHR-based implementation strategies for complex prescribing guidelines should consider both workflow integration and recipient expertise.

**Trial Registration:**

(ClinicalTrials.gov: NCT05351749).

**Supplementary Information:**

The online version contains supplementary material available at 10.1186/s13012-025-01478-9.

Contribution to the Literature

- Inappropriate prescribing of anticoagulant medications significantly increases the risk of adverse drug events, including life-threatening bleeding and thromboembolism
- Asynchronous notifications within the electronic health record are a promising strategy to increase collaboration between medication prescribers and clinical pharmacists
- There was no difference in the overall adoption of appropriate anticoagulant prescribing between notifications sent to prescribing clinicians or clinical pharmacists

Clinical pharmacists demonstrated greater nuance in the adoption of evidence-based prescribing than prescribing clinicians working independently of clinical pharmacists.

## Background

Overuse and errors in medication prescribing are common and lead to harm at both the patient and public health levels. For example, overuse of antibiotics leads to antibiotic-resistant organisms [1], while inappropriate prescribing of anticoagulants can lead to life-threatening thrombotic and/or bleeding complications [2]. Medication prescribing is primarily the responsibility of healthcare providers, namely physicians, nurse practitioners, and physician assistants. However, clinical pharmacists with specialized knowledge about appropriate medication prescribing are often not well integrated into medication prescribing workflows within most health systems. Furthermore, integrating highly trained clinical pharmacists into ambulatory prescribing workflows requires significant financial and organizational resources [3, 4]. In fact, many hospitals and health systems currently do not employ clinical pharmacists with anticoagulation expertise to work in primary care or anticoagulation stewardship clinics. Furthermore, these clinical pharmacists have expertise in medication management, but do not have prescribing authority in most healthcare systems. Therefore, evidence demonstrating the clear benefit of these clinical pharmacists would justify institutional investment in these experts to deliver ambulatory antithrombotic stewardship interventions and to coordinate with prescribing clinicians.

The proliferation of comprehensive electronic health records (EHR) enables the use of low-to-no-resource implementation strategies aimed at influencing clinician behavior. These include clinical decision support systems, Our Practice Advisory alert, and automated messaging, which can support evidence-based prescribing. However, the added benefit of integrating clinical pharmacists into the EHR-based strategy to influence evidence-based prescribing in the ambulatory setting is not as well understood [5]. Therefore, we aimed to compare two implementation strategies employing EHR-based notifications for potentially inappropriate prescribing to examine the change in effectiveness when clinical pharmacists were and were not specifically engaged. Furthermore, these two implementation strategies were designed to be non-interruptive to minimize workflow disruption, which contributes to clinician burnout.

Oral anticoagulant prescribing, particularly of the direct oral anticoagulants (DOACs), is an ideal case study for comparing low-resource automated EHR asynchronous notification to higher-resource clinical pharmacist engagement to correct potentially inappropriate prescribing. DOAC medications are currently the leading cause of adverse drug events leading to emergency department visits [6], in part due to the high rate of inappropriate prescribing [7, 8] that is linked to worse clinical outcomes [2]. In fact, these prescribing errors often develop *after* an initial prescription is written [9], due to changes in patients’ age, weight, and other relevant characteristics, which limits the ability of an automated EHR alert at the time of initial prescribing to impact a prescription that may become inappropriate after initially prescribed.

## Trial design

### Study overview

As part of a larger trial testing several strategies for reducing inappropriate DOAC prescribing, [10] we conducted a two-arm, cluster-randomized implementation trial of an automatic asynchronous EHR notification (i.e., in basket message) to be sent to either a DOAC-prescribing clinician or a clinical anticoagulation pharmacist to review potentially inappropriate DOAC prescribing. The goal of the study is to see which recipient of an automatic asynchronous EHR notification eventually leads to higher rates of changes to DOAC prescriptions. Following the receipt of a notifications, prescription changes were able to be made without a formal clinic visit, with patients notified typically either by patient portal messaging or through a telephone call. The study dates included May 4, 2023 – December 15, 2024. This study was reviewed by the University of Michigan Medical Institutional Review Board and approved with a waiver of informed consent for the prescriber and patient, given the study’s focus on improving standards of care and minimal risk. It is registered at ClinicalTrials.gov (NCT05351749).

### Participant eligibility

All DOAC prescriptions written in an ambulatory setting for adult patients (age 18 years or greater) who see Michigan Medicine clinicians with prescribing privileges were assessed to see if they followed evidence-based prescribing rules. Prescriptions written in the Emergency Department, inpatient setting, in a skilled nursing facility, and medication orders written by study team members were excluded [10]. Patients with multiple anticoagulants on their medication list were captured in a concurrent study focusing on the time of active prescribing and were not present in this study focused on the chronic phase of anticoagulant management [10]. Prescribers were notified of the ability to opt out of the trial through a system-wide electronic message at the beginning of the study. Prescriptions were deemed inappropriate—and prescribers were eligible for randomization—if they were found not to follow the Food and Drug Administration (FDA) package label instructions for appropriate dosing by indication or had a significant drug-drug interaction as outlined by the Anticoagulation Forum (see Supplemental Appendix A for full details). As this study focused on mitigating inappropriate chronic DOAC use rather than initial DOAC prescriptions (a focus of a different, concurrent study [10] also covered by this trial protocol), medication errors detected within 7 days of a new prescription were excluded. This study focused on medication errors that persisted or developed beyond 7 days after a prescription was written.

After the trial was initiated, a protocol amendment was made to exclude patients being treated with nirmatrelvir/ritonavir for COVID-19 infection. This combination medication is known to interact with DOAC medications; however, a system-wide process was implemented, independent of our study, to instruct patients on how to adjust their anticoagulant while taking nirmatrelvir/ritonavir. As such, these instances of inappropriate DOAC prescribing due to a drug-drug interaction were excluded from the study.

### Randomization

Prescribers of eligible prescriptions were randomized at their first instance of the EHR tool automatically detecting the inappropriate DOAC prescriptions. Randomization occurred with equal probability to one of two types of asynchronous EHR notifications: (1) prescriber-directed messages or (2) clinical pharmacist-directed messages. *Prescriber-directed messages* were asynchronous notifications that were routed to the prescriber for review when an existing DOAC prescription(s) met criteria for being potentially inappropriate according to the FDA package label instructions. *Pharmacist-directed messages* were asynchronous notifications that were routed to the anticoagulation clinic pharmacist’s EHR in basket when an existing DOAC prescription met criteria for being potentially inappropriate according to the FDA package label instructions. The content of the two asynchronous messages was nearly identical (Supplemental Appendix B). Any pharmacist-directed message would require prescriber collaboration, as clinical pharmacists do not have independent prescribing authority at Michigan Medicine. A permuted block randomization was used via computer scripts built into the EHR for prescriber-level randomization. Randomization was stratified by trainee vs. primary care vs. specialists. Prescribers may have multiple (existing) inappropriate prescriptions during the trial. Randomization assignment for the prescriber, once randomized, remained consistent throughout the trial. Prescribers were aware of their assignment, while the analytic team was blinded to randomization.

### Data collection

Through the asynchronous Our Practice Advisory framework in the Epic® EHR, all prescriptions of DOACs for non-hospitalized patients were reviewed and measured against new data entered into the EHR over the preceding 24 h (e.g., laboratory results, changes in age and weight, new or changed prescriptions, etc.), daily at 8 AM. If any of the newly entered values resulted in the prescription no longer following the established prescribing guidelines, the prescription was flagged as inappropriate, and a notification was sent according to the randomization scheme outlined above.

Following an EHR upgrade in May 2024, an unanticipated change limited the number of potential DOAC prescription errors identified by the asynchronous framework. Specifically, any new medication prescriptions after that upgrade were not able to be evaluated by the Our Practice Advisory framework. Therefore, the number of notifications sent after May 2024 was notably lower than before that time (Supplemental Appendix C).

## Hypotheses and outcome measures

Given that the clinical pharmacists in our anticoagulation clinic were explicitly tasked with managing DOAC medications and possess extensive anticoagulant expertise, and most prescribers have numerous competing priorities and less specific expertise with DOAC medications, we hypothesized that clinical pharmacist engagement would result in a higher proportion of changes than prescriber-directed automated notifications. In pre-specified exploratory analyses, we examined potential moderators to understand whether certain types of prescribers and/or patients would benefit more from clinical pharmacist engagement.

### Primary outcome: prescriptions changed

The primary outcome, defined in our protocol paper as *adoption*, was the proportion of existing medication notifications that resulted in any prescription change within 7 days from delivery of the notification. Each notification was considered to have resulted in a “change” if any change was made in terms of dosage, frequency, and/or medication being prescribed when compared to the DOAC medication that triggered the notification. Each notification was coded as changed or not changed by comparing the DOAC prescription triggering the notification message with the medication prescription details at 7 days following the notification. Any discontinuation of a medication that persisted until day 7 following the notification was also considered a change. The data were retrieved from the University of Michigan EHR through custom-built reports. When the data were unclear for determining the outcome, cases were reviewed manually by a study team member (MF), who was blinded to assignment and did not participate in further analysis.

### Secondary and exploratory outcomes

While our primary outcome was the proportion of inappropriate prescriptions changed within 7 days (*Adoption*), prescriptions changed within 14 or 30 days were also examined as a post-hoc secondary outcome. These longer windows, which were not included in our protocol, were added after the trial began based on feedback from the clinical pharmacists, who noted that it often required more than 7 days to review a chart and communicate with prescribing clinicians. Per protocol, we examined several exploratory outcomes based on the RE-AIM implementation evaluation framework [10, 11]. These included the proportion of DOAC-treated patients and DOAC-prescribing providers who triggered a notification (*Reach*); the proportion of prescribers that implemented at least one prescription change during the study period (a secondary measure of *Adoption*); the percentage of changes made as recommended in the notification (*Implementation fidelity*); and changes in the proportion of prescription changed during the 20 month study period (*Maintenance*). To better understand resource requirements of the different strategies, a sample of the clinical pharmacist workload was collected. Specifically, pharmacists working in the UM Anticoagulation service were asked to track the time they spent responding to and resolving the DOAC notifications associated with this study over a three-week period in March 2024.

### Other patient and prescriber-level data, including moderators

Patient characteristics at the time of notification, including gender, age, weight, presence of impaired renal function (based on creatinine clearance level [CrCl] as calculated by Cockcroft-Gault equation [12] using actual body weight and actual serum creatinine values), and specific diagnosis (venous thromboembolism [VTE] vs. atrial fibrillation [AF]) were collected. Prescriber role (trainee vs. primary care vs. specialist), provider type (physician, physician assistant, nurse practitioner, or resident), and frequency of DOAC prescriptions by the provider prior to the study were also collected.

## Statistical analysis

### Primary analyses

The primary analysis compared the main effect of Prescriber-directed messages versus Pharmacist-directed messages on our primary outcome, the proportion of inappropriate existing DOAC prescriptions that were changed in 7 days, using mixed-effects logistic regression models. Models were run with the prescriptions as the unit of analysis, with fixed effects for the notification type, stratification variables (trainee vs. primary care vs. specialists) and an unstructured covariance matrix for residual errors. A prescriber-level random effect was included to account for prescription clustering within prescribers. Sensitivity analyses, including a patient-level random effect, were also conducted.

While the primary analysis focused on changes made within 7 days of notification delivery, changes made within 14 days and 30 days were further examined as post-hoc secondary outcomes to consider cases that took longer to make prescription changes. A post-hoc subgroup analysis was also conducted. After the trial was completed, it was noted that both prescribers and pharmacists frequently did not make a prescription change when a patient was near a threshold for inappropriate dosage (e.g., weights fluctuating around the threshold, age changing soon, etc.). The subgroup analysis tested whether differences in prescriber- vs. pharmacist-directed notifications were more apparent for a subset of inappropriate prescriptions that met an expanded criteria (i.e., farther from the border level) for values of patients’ serum creatinine, creatinine clearance, weight, or ages (Supplemental Appendix Table D1). For each scenario of notifications (i.e., specific DOAC medication, dose too high/low), we defined and applied these expanded criteria, and then reran our primary analysis as above, using only the subset of cases that met this expanded definition of the criteria. Sensitivity analyses, with the changes made in 14 days and 30 days, were also conducted on the same subset of cases.

### Secondary and exploratory analyses

Reach, adoption, effectiveness, and implementation fidelity outcomes were assessed descriptively. Implementation fidelity was also assessed for differences between prescriber- and pharmacist-directed messages. Maintenance, or an over-time trend of the prescriptions changed (overall and by notification type), was assessed by adding a fixed effect for time and a time by treatment interaction to the model. Resource utilization was assessed as the average minutes spent per notification, calculated by dividing the total time pharmacists spent on DOAC notifications for this study (in minutes) by the number of notifications pharmacists worked on during a three-week period.

### Moderators

Moderators for the effect of the different types of notifications were further explored: two prescriber-level moderators (specialty vs. primary care providers, number of DOAC prescriptions before the study divided into tertiles), four moderators of patient characteristics (patient age, presence of impaired renal function, polypharmacy (five or more prescribed medications), and specific diagnosis), and two moderators about prescriptions (apixaban vs. rivaroxaban, dose too high vs. too low) were examined. Post-hoc moderator of near vs. farther from cut-off threshold values was also examined. While our protocol specified examining patient-level moderators by diagnosis, this was not completed, given that only 2% of notifications were triggered for VTE. Given the exploratory nature of these analyses, significance was assessed by examining 90% confidence interval [CI].

### Power and sample size

Power calculations have been previously described [10]. Assuming 600 patients across 300 prescribers, a prescriber-level intraclass correlation (ICC) of 0.1, and a type-1 error rate of 5%, we had 94% power to detect the difference in the proportion of prescription changes of 0.40 vs. 0.55 (risk ratio of 1.45) for prescriber-directed vs. pharmacist-directed messages.

## Results

### Notifications and participants

**Fig. 1:**
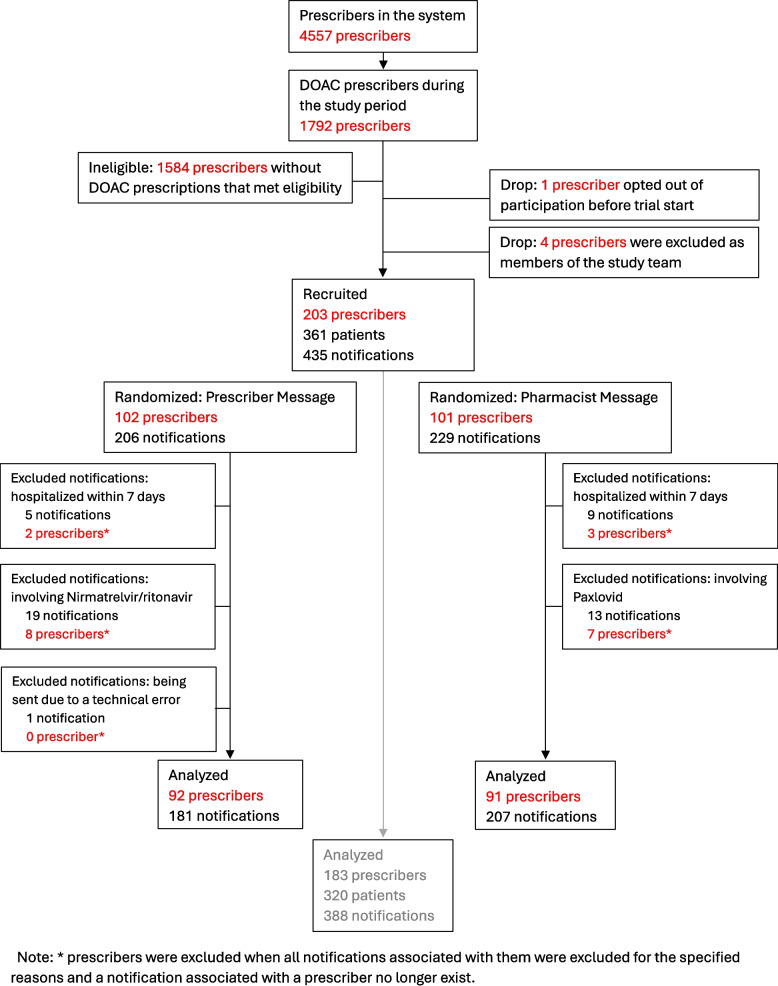
Consort Diagram

Five prescribers opted out of study eligibility or were excluded prior to study start (Fig. 1). During the 20-month study period, 435 notifications were sent for inappropriate prescribing among 203 prescribers.

#### Reach

Among all 1,792 DOAC prescribers eligible for the study, 203 (11.3%) prescribers triggered notifications and were randomized. Among 20,470 DOAC-prescribed patients, 361(1.8%) patients triggered one or more notifications. The median number of notifications per month was 26 (interquartile range [IQR] = 11–30). Due to a programming error introduced during an EHR upgrade in May 2024, the number of notifications declined significantly thereafter: the median number of notifications per month was 28 (IQR = 26–32) between May 2023 and May 2024, while it was 9 (IQR = 4–11) between June 2024 and December 2024 (additional details and implications of this error are included in the Discussion section, below).

#### Resource utilization

Clinical pharmacists spent an average of 7.9 min (standard deviation [SD] = 4.0; range = 3–20 min) responding to and/or resolving each notification for this study. There was no difference in the median time from notification to prescribing change in the two arms (median = 2 days for both groups; Supplemental Appendix Table E4).

Of the 435 notifications, 47 were excluded from the analyses due to hospitalization within 7 days of the notification, involving nirmatrelvir/ritonavir, or a technical error; 388 notifications among 183 prescribers were included in the analyses (Fig. 1).

Of 388 notifications included in the analyses, 290 (74.7%) were related to apixaban and 98 (25.3%) to rivaroxaban; 5 (1.3%) involved drug-drug-interaction contraindications (Table 1). Notifications were initiated for 249 (64.2%) cases where the values of patients’ age, weight, and/or renal function were close to the cut-off threshold value for appropriate dosing and 139 (35.8%) cases where the values were farther from the cut-off threshold for appropriate dosing (thresholds defined in Supplemental Appendix Table D1).

**Table 1:** Notification characteristics by notification randomization (*n* = 388)

		**All (*n* = 388)**	**Prescriber message (*n* = 181)**	**Pharmacist message (*n* = 207)**
		*n* (%)	*n* (%)	*n* (%)
**Reasons for Notification**	**DOAC**			
Dose too low	Apixaban	185 (47.7)	100 (55.2)	85 (41.1)
	Rivaroxaban	37 (9.5)	19 (10.5)	18 (8.7)
Dose too high	Apixaban	102 (26.3)	39 (21.5)	63 (30.4)
	Rivaroxaban	55 (14.2)	21(11.6)	34 (16.3)
Contraindication – Afib	Apixaban	0 (0.0)	0 (0.0)	0 (0.0)
	Rivaroxaban	4 (1.0)	0 (0.0)	4 (1.9)
Contraindication – VTE	Apixaban	3 (0.8)	2 (1.1)	1 (0.5)
	Rivaroxaban	2 (0.5)	0 (0.0)	2 (1.0)
**Near-Threshold Values (Age, Weight, Renal Function)**				
Near the threshold level		249 (64.2)	121 (66.9)	128 (61.8)
Outside of the border level		139 (35.8)	60 (33.1)	79 (38.2)

Of 183 prescribers included in the analysis, 12 (6.6%) were trainees, 108 (59.0%) were primary care clinicians, and 63 (34.4%) were specialist clinicians. The majority of prescribers were physicians (71.6%), followed by nurse practitioners (16.9%), residents (6.6%) and physician assistants (4.9%) (Table 2). Relative to the population of DOAC prescribers that were not included in the study, these 183 prescribers were comprised of more primary care clinicians (59% vs. 19%) and fewer residents (7% vs. 37%). Perhaps unsurprisingly, they also had a much higher volume of DOAC prescriptions in the pre-trial period (median = 26 vs. 1) (Supplemental Appendix Table E1).

**Table 2:** Provider-level characteristics by notification randomization (*N* = 183)

	**All (*N* = 183)**	**Prescriber message (*N* = 92)**	**Pharmacist message (*N* = 91)**
	*n* (%)	*n* (%)	*n* (%)
Role			
Resident	12 (6.6)	7 (7.6)	5 (5.5)
Primary Care	108 (59.0)	53 (57.6)	55 (60.4)
Specialty	63 (34.4)	32 (34.8)	31 (34.1)
Provider Type			
Nurse Practitioner	31(16.9)	14 (15.2)	17 (18.7)
Physician	131 (71.6)	65 (70.7)	66 (72.5)
Physician Assistant	9 (4.9)	6 (6.5)	3 (3.3)
Resident	12 (6.6)	7 (7.6)	5 (5.5)
DOAC prescriptions pre-trial			
Fewer (1st tertile)	62 (33.9)	31 (33.7)	31 (34.1)
Medium (2nd tertile)	60 (32.8)	33 (35.9)	27 (29.7)
More (3rd tertile)	61 (33.3)	28 (30.4)	33 (36.3)

Of the 183 prescribers, 94 (51.4%) had prescriptions triggering a single notification during the study period, and 89 (48.6%) had prescriptions triggering multiple notifications. Of the remaining 89 prescribers whose prescriptions triggered multiple notifications, 63 prescribers had single notifications triggered by multiple patients, five prescribers had multiple notifications for a single patient, and 21 prescribers had patients that triggered both single and multiple notifications. The number of unique patients per prescriber ranged from 1 to 7, with a median of 1 (IQR 1–2).

Patient-level characteristics were balanced between study arms (Table 3). Among the 320 patients with notifications during the trial period, 23 (7.2%) had notifications sent to multiple prescribers and 11 patients (3.4%) triggered notifications for at least one provider randomized to each study arm; these patients were counted in both arms when summarizing patient-level characteristics.

**Table 3:** Patient-level characteristics (*n* = 388)

	**All (*n* = 388)**	**Prescriber message (*n* = 181)**	**Pharmacist message (*n* = 207)**
	*n* (%)	*n* (%)	*n* (%)
Age: Mean (SD)	83.1 (7.9)	82.90 (8.7)	83.2 (7.3)
Age > 70	368 (94.9)	168 (92.8)	200 (96.6)
Weight (kg): Mean (SD)	77.06 (19.6)	78.2 (19.1)	76.1 (20.1)
Female	199 (51.3)	84 (46.4)	115 (55.6)
Polypharmacy	337 (86.9)	155 (85.6)	182 (87.9)
Impaired renal function (CrCl ≤ 60)	340 (87.6)	157(86.7)	183 (88.4)
VTE (vs. Afib.)	9 (2.3)	2 (1.10)	7 (3.38)
Apixaban (vs. Rivaroxaban)	292 (75.3)	141 (77.9)	151 (73.0)
Notification			
*Dose too low*	222 (57.2)	119 (65.8)	103 (49.8)
*Dose too high*	157 (40.5)	60 (33.2)	97 (46.9)
*Contraindicated*	9 (2.3)	2 (1.1)	7 (3.4)

All values are *n*(%) unless otherwise specified

*SD* Standard deviation, *CrCl* Creatinine clearance

### Overall prescription changed

#### Adoption

38.3% of prescribers (N = 70/183) made a change in at least one of their existing inappropriate DOAC prescription notifications. Overall, 90/388 (23.2%) existing inappropriate DOAC medication notifications resulted in any prescription change within 7 days from the notification being sent. The number of notifications and changes made each month is shown in Supplemental Appendix C. Expanding the time frame from the pre-specified 7-day range resulted in changes to inappropriate DOAC prescriptions in 104/388 (26.8%) notifications within 14 days, and 117/388 (30.2%) notifications within 30 days. Seven patients switched anticoagulant medications: five from one DOAC to another and two from a DOAC to a non-DOAC medication (Supplemental Appendix Table E5).

#### Maintenance

Over the 20-month study, the percentage of notifications in either arm that resulted in a prescription change increased slightly but not statistically significantly over time, increasing from an average of 19% in Month 1 to 34% in Month 20 (estimated marginal effect of time = 0.8% per month, 95%CI = −0.2–1.7% per month). 

#### Implementation fidelity

Of the 90 notifications that resulted in a prescription change, 88 (97.8%) resulted in a clinically appropriate medication change; 84 (93.3%) were changes recommended by the notification, and four (4.4%) were changes that differed from the notification recommendation but were still adjudicated as clinically appropriate. Two notifications (2.2%) resulted in a clinically inappropriate medication change despite the text of the notification recommending clinically appropriate medication changes. Both of these cases involved patient self-discontinuation of the medications in prescriber-directed messages. With respect to fidelity of the notifications, two notifications (0.5%), both in the prescriber-directed arm, were found to have been sent in error. When the primary analyses were run without these cases in a sensitivity analysis, our findings remained unchanged.

### Primary outcome: comparison of prescriber- vs. pharmacist-directed notification

**Fig. 2:**
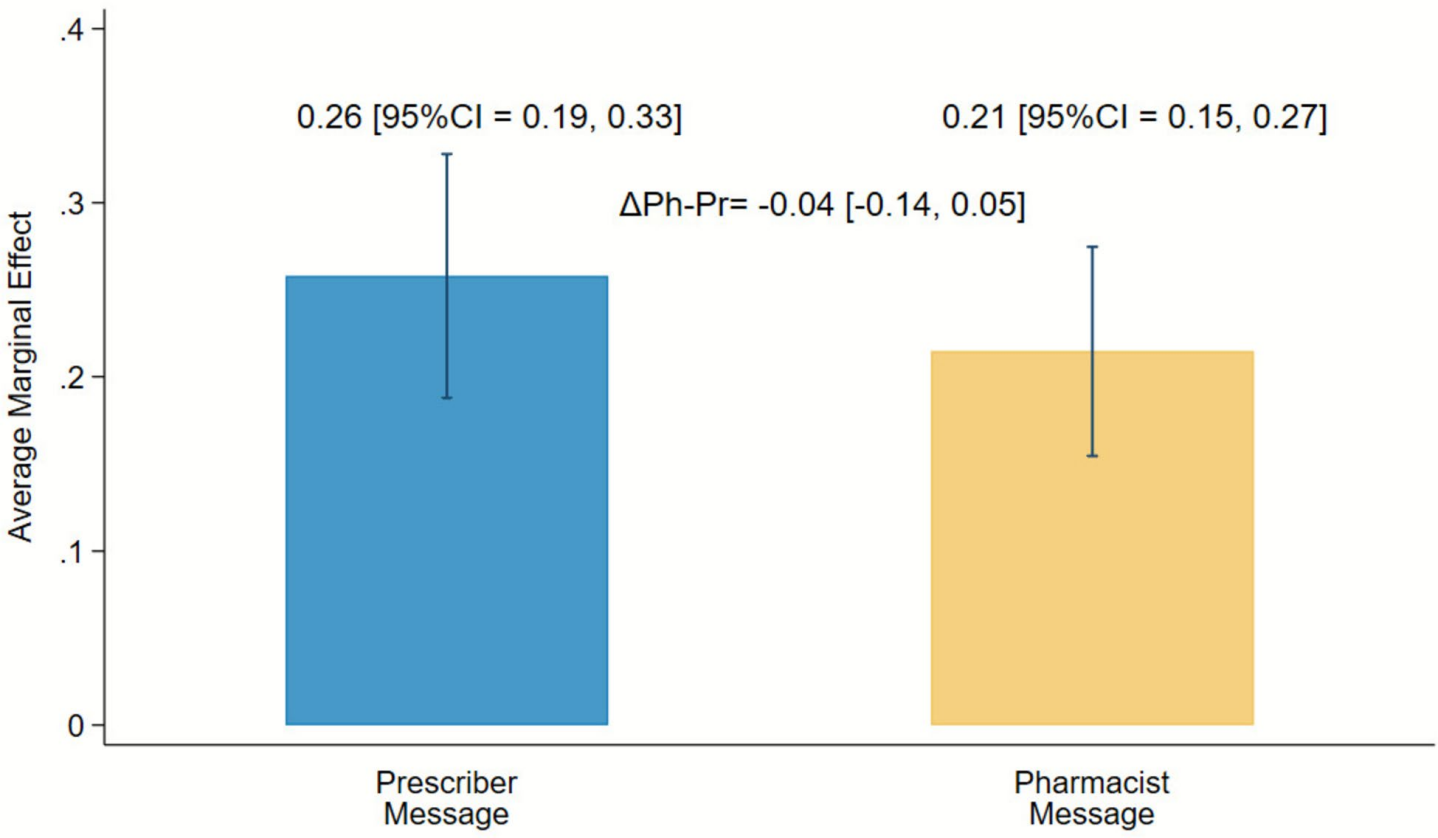
Change to DOAC prescription by notification recipient: average marginal effect by study arm

The estimated percentage of inappropriate DOAC prescriptions that resulted in a change within 7 days was 26% for the prescriber-directed messages and 21% for the pharmacist-directed messages; this difference was not statistically significant (difference = 4% lower for pharmacists versus prescribers, 95% CI = 14% lower to 5% higher, p = 0.36; Fig. 2) and contrary to our hypothesis that involvement of pharmacists would lead to changes in more prescriptions. Sensitivity analyses, including a patient-level random effect to account for multiple notifications per patient and expanding the outcome time frame to 14 and 30 days, found similar, non-statistically significant differences between the two arms (Supplemental Appendix Figure E1, Tables E2 and E3).

### Subgroup analysis for prescription changes

**Fig. 3:**
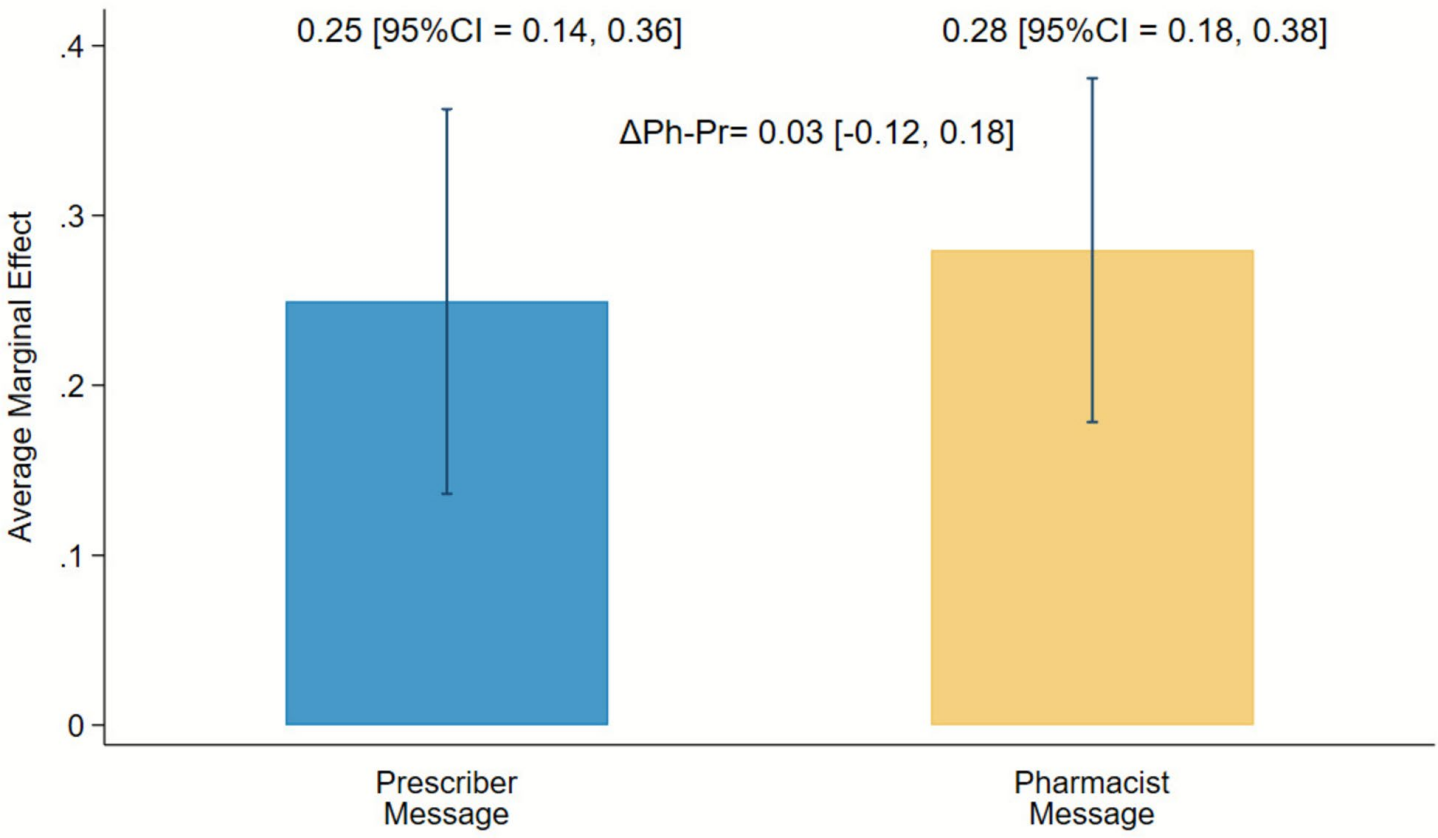
Subgroup analysis of scenarios further from cut-off threshold values for age, weight, and/or renal function

A post-hoc subgroup analysis assessed the proportions of prescriptions changed across study arms for those that were farther away from the cut-off threshold for age, weight, and/or renal function, consisting of 139 (35.8%) notifications. Of 139 notifications, 43.2% (n = 60) were prescriber-directed messages and 56.8% (n = 79) were pharmacist-directed messages. DOAC prescriptions were changed for 25% of the prescriber-directed and 28% of the pharmacist-directed messages in this cohort (difference 3% higher for pharmacists versus prescribers, 95% CI = 12% lower to 18% higher, Fig. 3). Similar trends were seen with prescription changes at 14 and 30 days (Supplemental Appendix Figure D1, Table D2).

### Moderator analyses

**Fig. 4:**
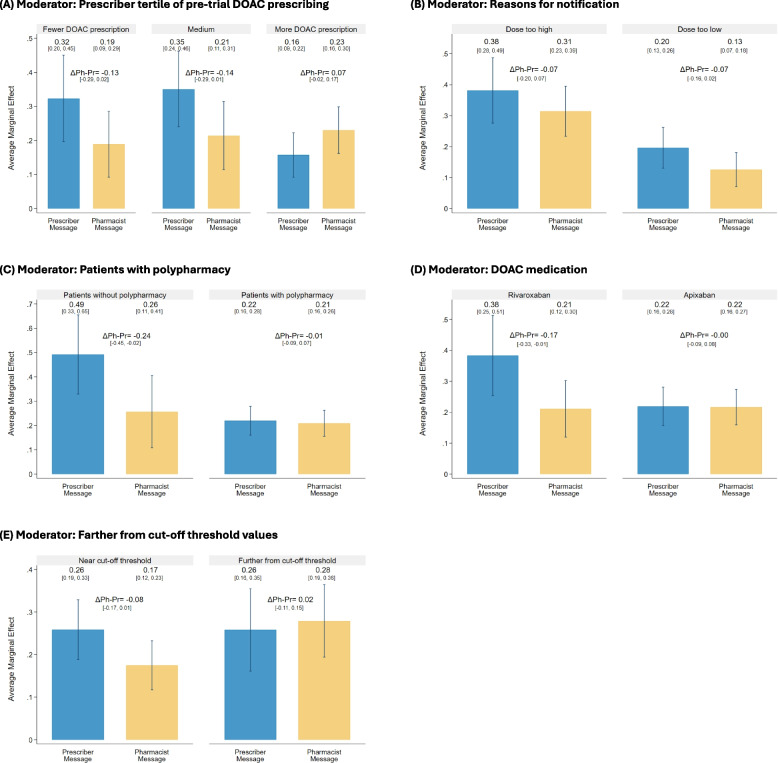
Moderator analyses: Change to DOAC prescription by notification recipient and moderators

The only statistically significant moderator of treatment effects was prescriber pre-trial DOAC prescribing volume (Fig. 4A). Medication changes occurred less frequently when notifications were sent to prescribers with a high volume of prior DOAC prescribing (16%) as compared to those with low volume (32%) and medium volume (35%). This contrasts with the notifications sent to pharmacists, where the proportion of DOAC changes was similar regardless of the prescriber’s prior DOAC prescription volume (19–23%). This results in a difference in differences across prescriber- and pharmacist-directed notifications of 21% (90% CI 3% to 39%).

The moderator analyses also show that in both prescriber- and pharmacist-directed messages, fewer changes were made for DOAC dose that were too low (i.e., increasing dose is recommend) compared to when the dose was too high (difference for prescribers: 19% lower, 90% CI 6–31% lower; difference for pharmacists: 19% lower, 90% CI 9–28% lower) (Fig. 4B). In addition, prescriber-directed notifications resulted in fewer changes when a patient had polypharmacy (five or more prescribed medications) compared to no polypharmacy (22% vs. 49%, difference of 27% lower, 90% CI 10–44% lower) (Fig. 4C). Lastly, prescribers also made fewer medication changes for apixaban than rivaroxaban (22% vs. 38%, difference of 16% lower, 90% CI 2–31% lower (Fig. 4D). A post-hoc moderator analysis shows that pharmacist-directed messages were more likely to result in a prescription change when clinical values were farther from versus closer to the dosing thresholds (28% vs. 17%, difference of 10% higher, 90% CI 1%—20% higher). The full results of the moderators analysis are in Supplementary Appendix Table F1).

## Discussion

This study found that the use of EHR-based, asynchronous notifications resulted in prescription changes for inappropriate DOAC use in just under one in four clinical scenarios among both prescribers and pharmacists. Although we hypothesized that notifications would be more effective at prompting changes to inappropriate prescriptions when they went to DOAC-specialty pharmacists rather than prescribers, there was no statistically significant difference in these rates. However, when prescribing changes occurred after an EHR-based notification, they were overwhelmingly (97.8%) aligned with best clinical practice.

This overall rate of medication change is likely related to the clinical scenario and method by which it is delivered. In one recent study of drug-drug interaction alerts, medication prescription changes occurred 18.6% of the time when the alert was interruptive and only 8.4% of the time when the alert was non-interruptive [13]. Those rates are even higher than older studies of drug-drug interaction alerts using interruptive alerts, showing medication change rates of 1.5–8.4% [14, 15]. In contrast, another recent study examining medication alerts that recommended lower-cost alternatives resulted in prescription changes in 18–47% of cases, depending on the specific medication [16]. This is mainly in line with other studies showing rates of medication alert override exceeding 50% [17, 18]. In our trial comparing non-interruptive, asynchronous notifications for both incorrect dosing and drug-drug interactions sent to two different parties, only 23% led to a prescription change within seven days across both arms. That rate did increase to 30% when the timeframe for change was extended to 30 days. However, this is not meaningfully higher than previously reported literature.

Several explanations for an overall low rate of change are plausible. First, the academic medical center within which this study took place has been at the forefront of addressing inappropriate DOAC prescribing, including the establishment of a pre-existing program for identifying inappropriate DOAC prescriptions that had been operating for three years prior to trial start. As such, the rate of inappropriate DOAC prescribing is notably lower in our cohort (2%) than in other cohorts (average 20%) [19]. Therefore, the identified cases may also have been more challenging to address or correct. This is especially important when considering that binary guideline recommendations, which apply to the majority of clinical cases, may not always represent the best medical decisions for a minority of complex patient situations [20]. Second, this study specifically examined changes to existing (rather than new) prescriptions for a medication that is often taken long-term. The criteria used by the EHR to trigger an alert are complex and fluctuating, sometimes from day to day (e.g., weight, renal function). These factors may contribute to a lower overall rate of medication change when notifications were provided. More importantly, however, they suggest a need for more nuanced implementation science work to understand how and when to trigger non-interruptive EHR alerts that encourage evidence-based prescribing, particularly for long-term use medications with complex and dynamic criteria. For example, the use of clinically informed, adaptive decision rules that only trigger notifications after someone has crossed a changeable criteria threshold by a certain amount or a certain number of times might help maximize alert effectiveness and minimize the burden and negative externalities of alert fatigue. Additionally, the ability for specific providers or institutions to customize alert cutoffs may help improve adoption. Integration of artificial intelligence models to prioritize notifications that maximize the likelihood of a prescription change may further reduce clinician alert fatigue while maintaining overall effectiveness. Additionally, medication guidelines may benefit from distinguishing between initial prescribing rules and longer-term follow-up rules (i.e., when to adjust doses based on fluctuating renal function).

We also, somewhat surprisingly, found no support for the primary hypothesis of this implementation trial, which was that intentional integration of clinical specialty pharmacists into an asynchronous medication notification workflow would increase the rate of prescription changes versus sending notifications to prescribers (primary outcome of adoption). Rather, unlike prior studies demonstrating significant improvements for pharmacist-involved management of DOACs in inappropriate prescribing as compared to usual care [21–23], rates of change for both groups were very similar in our trial. One potential explanation for this is the level of prior integration of the DOAC specialty pharmacists within this academic medical center. It is possible that prescribers were already accustomed to engaging DOAC pharmacists in determining which DOAC medication, dose, and frequency correctly followed evidence-based prescribing. Thus, regardless of the initial recipient of the notification (prescriber or pharmacist), the decision-making process triggered by notifications engaged both parties collaboratively and did not differ significantly across arms. Future analyses will utilize EHR and patient chart data to examine the decision-making process across both arms and determine whether collaborative decision-making for prescription changes was indeed the norm. Another potential explanation is that the prescriber-directed notifications were sufficiently detailed to provide a similar level of clinical decision support as that of a well-trained anticoagulation pharmacist. A more traditional notification with less detail may have resulted in fewer prescription changes in the prescriber-directed arm [24].

Although no significant differences were found across the study arms, there may still be reason to believe that the DOAC pharmacists are better recipients of these notifications, even if they require more overall resources to deploy than a prescriber-directed notification. Certainly, evidence of notification fatigue and related negative effects (e.g., burnout) for prescribers is robust [25–27], and any efforts to diminish (even asynchronous) EHR alerts that are consistent with upholding care quality should be considered and analyzed for their overall resource requirement. This is particularly relevant when 70% or more of the EHR alerts result in no clinical change. Furthermore, it is notable that the percentage of DOAC changes made by pharmacists was significantly lower for notifications where clinical values were closer to dosing thresholds (17%) compared to when the values were farther from the thresholds (28%). Similar differences were not found for prescribers, who changed prescriptions both near and far from the thresholds at a largely similar rate. This suggests that the clinical pharmacists were highly attuned to nuances in appropriate DOAC prescribing and recognized the importance of individualizing decision-making for patients, even around adherence to clinical guidelines, based on individual patient characteristics and/or histories (e.g., fluctuating weight, renal function). Analyses showed that clinical pharmacists also changed inappropriate prescription rates equally regardless of the presence of polypharmacy. This is in contrast to prescribers who were less likely to change inappropriate DOAC prescriptions when patients had polypharmacy as compared to fewer than 5 total prescriptions. The consistent rate of medication change by the clinical pharmacists suggests the value of their expertise in navigating the implementation of complex prescribing guidelines for (likely) medically complex patients, even if the primary adoption outcome was not different in this study. This study also found that the resources required to integrate pharmacists into the DOAC notification and change workflow were minimal, averaging only 8 min per notification.

This trial exemplified both the potential impact and pitfalls of designing EHR-based implementation strategies and trials, particularly for extended periods and at a systems level. Although we had a key system-lead EHR stakeholder as a Co-Investigator on the study team, we still encountered technical issues that hampered the fidelity and potential impact of this study. Specifically, most EHR systems undergo regular maintenance and updates to incorporate new features and comply with new regulations. In our case, one of these updates inadvertently led to a programming error that significantly reduced the number of patients identified as eligible for the notifications. Although our study team had piloted our notifications system and also had robust monitoring processes in place, the error persisted for a period of approximately seven months because (a) notification rates varied naturally month-to-month, and thus several months of data were necessary to confirm that an error likely existed, (b) there was some delay (up to four weeks) in the study team gaining access to data for monitoring, and then (c) the cause of the error had to be identified and fixed by members of health system EHR team. Despite this error, however, our study was able to reach a large patient population, screening more than 20,000 patients and nearly 1800 prescribers for possible inclusion over 18 months. However, as more implementation trials leverage EHR-based implementation strategies and data, further guidance on best practices for robust implementation of longer-term trials should be developed, specifically incorporating health system EHR executives as part of the team, establishing transparent communication strategies around systems updates and data monitoring expectations, and developing protocols for how and on what timeframe intervention fixes due to system updates or changes will be addressed. Furthermore, the effective use of any EHR-based implementation strategies, whether research- or practice-based, should include ongoing information technology monitoring and assistance to address unforeseen issues.

We acknowledge several important limitations to this study. First, as discussed above, this trial was conducted in a single center with a well-established, pre-existing antithrombotic stewardship program and a largely integrated network of prescribing clinicians. We anticipate that a similar study conducted in a healthcare system with different levels of network integration and/or levels of clinical pharmacist support may find different results. Second, as noted above, due to a programming error that hampered notifications delivery during the final seven months of the trial, we sent fewer notifications to fewer prescribers than anticipated. While we do not believe this error introduced any bias into our intervention in terms of patients included/excluded or had any differential impacts on study arms, it did leave us underpowered relative to initial power calculations. Third, this trial was conducted concurrently with a second EHR-based implementation trial that evaluated the comparative effectiveness of two different EHR-based “nudges” (or synchronous alerts) for decreasing new inappropriate DOAC prescriptions [10]. Many of the prescribers randomized here were also randomized as part of this trial and thus received those alerts. Furthermore, one of these alerts provided prescribers with the option to consult a DOAC pharmacist to make the prescription change. This may have increased prescribers baseline propensity to either act on the notification and/or consult with the DOAC pharmacists. Finally, this project relied on custom code written within the EHR to perform real-time randomization and generate the content of the notification messages that met our users’ needs [24]. Relying on custom code to generate these two aspects of the study may limit external generalizability in other systems without high level information technology support.

## Conclusion

This trial, conducted in a large academic medical healthcare system, demonstrated the potential impact of an EHR-based, asynchronous notification strategy for implementing evidence-based medication prescribing by engaging specialty clinical pharmacists in the decision-making workflow. We found that both pharmacists and prescribers changed prescriptions about one-quarter of the time and that changes were overwhelmingly in line with evidence-based prescribing guidelines.

This trial raises important questions about when pharmacists are most effective in care pathways focused on medication management, as well as how we can best implement (often complex) prescribing guidelines through the EHR. While pharmacists did not make changes to identified inappropriate DOAC prescriptions more often than prescribers, subgroup and moderator analyses suggest a more nuanced application of the guidelines than that of prescribers. Further assessment is necessary to understand whether sending notifications to different parties resulted in different (i.e., less collaborative) decision-making processes around patient-specific application of the guidelines. Furthermore, while the technical skills for delivering EHR notifications are now well-established in most healthcare systems, the science for understanding when and to whom to best deliver those notifications remains underdeveloped—particularly for implementing complex interventions and guidelines. While these results have shed some light on how often these notifications proved effective, we will also examine data to understand more specifically why changes were not made, and to hopefully inform more nuanced EHR-based implementation of prescribing guidelines for long-term DOAC use, both to ensure optimal patient care and minimize notification fatigue and burden.

## Supplementary Information

Supplementary Material 1.

## Data Availability

Data from this manuscript is available from the corresponding author upon reasonable request. This work is registered at ClinicalTrials.gov (NCT05351749), https://clinicaltrials.gov/study/NCT05351749
