# Supplementary material for: Implementing prescriber-pharmacist collaboration to improve evidence-based medication prescribing using asynchronous, non-interruptive electronic health record notifications
Source: Implement Sci. 2025 Dec 15;21:15. doi: 10.1186/s13012-025-01478-9 (PMC12914967; doi:10.1186/s13012-025-01478-9)
Supplement: Supplementary file 1 — Supplementary Material 1. [file 13012_2025_1478_MOESM1_ESM.docx]

**Appendix A.**

**FDA Guidelines and Drug-Drug Interaction Chart**

FDA guidelines:

For Apixaban and Rivaroxaban, decision rules dictating appropriate prescriptions were based on the FDA-issued labeling information (package insert information). FDA labeling information was reviewed for relevant changes every 6 months to ensure the accuracy of decision rules.

Package insert information is available from the FDA. For example: apixaban <https://www.accessdata.fda.gov/drugsatfda_docs/label/2012/202155s000lbl.pdf>

Rivaroxaban

<https://www.accessdata.fda.gov/drugsatfda_docs/label/2021/215859s000lbl.pdf>

Drug-Drug Interactions were selected from the Anticoagulation Forum’s Rapid Resource on Direct Oral Anticoagulant Drug-Drug Interaction Guidance:

<https://acforum.org/web/resource_files/1742403529-1600.pdf>

**Appendix B.**

**Sample Notification Message**

**
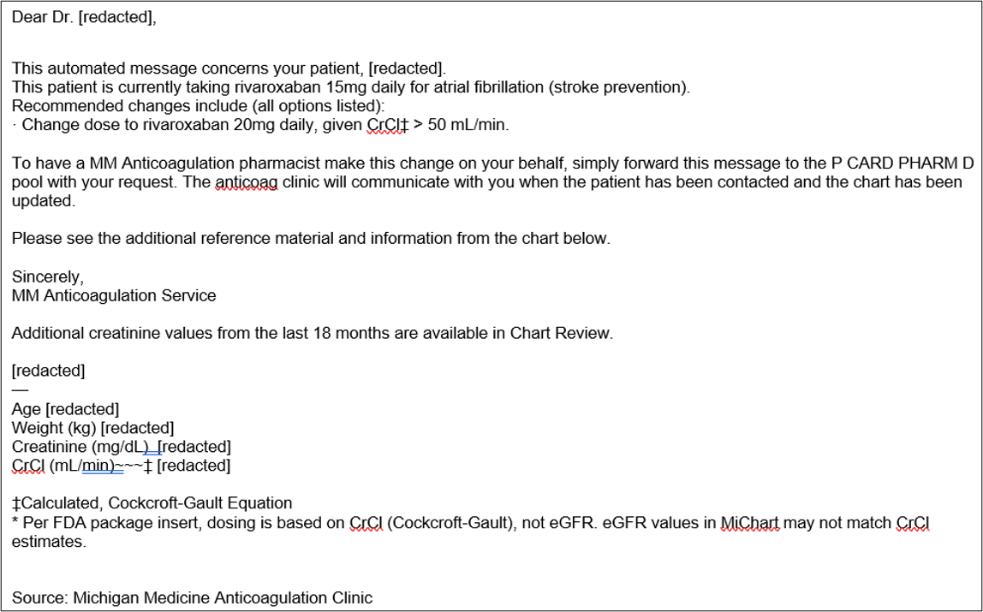
**

**Appendix C.**

**Figure C1. Number of notifications and changes made over time**

**
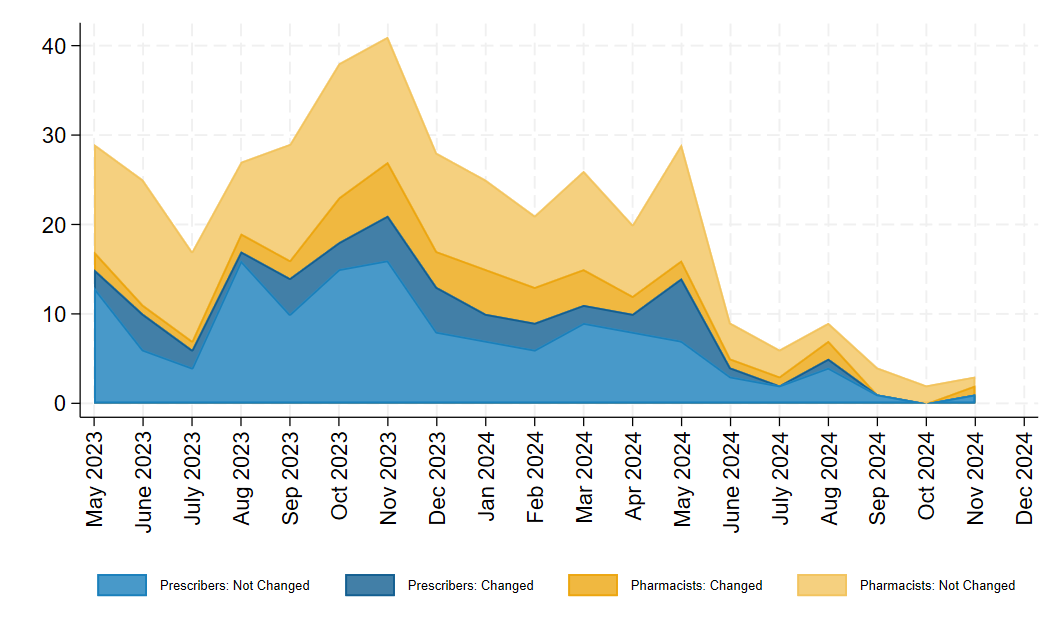
**

This stacked graph represents the total number of notification messages sent per month. Those in darker shades resulted in a medication prescription change, while the lighter shaded colors represent those that did not have a medication change within 7 days of the notification message being sent.

**Appendix D. Post-hoc, subgroup analysis**

**Table D1. Standard and Adjusted Criteria for Atrial Fibrillation Dose Adjustment**

|  | | **Standard Criteria** | **Criteria for “Further” from threshold value** |
| --- | --- | --- | --- |
| **Apixaban 5mg 🡪 2.5mg** | **Age** | ≥80 | ≥80 |
|  | **Weight** | ≤60 kg | ≤55 kg |
|  | **Serum Creatinine** | ≥1.5 mg/dL | ≥ 1.7 mg/dL |
|  |  |  |  |
| **Apixaban 2.5mg 🡪 5mg** | **Age** | <80 | <80 |
|  | **Weight** | >60 kg | >65 kg |
|  | **Serum Creatinine** | <1.5 mg/dL | < 1.3 mg/dL |
|  |  |  |  |
| **Rivaroxaban 20mg 🡪 15mg** | **Creatinine Clearance (Cockcroft Gault Equation)** | ≤50 ml/min | ≤45 ml/min |
|  |  |  |  |
| **Rivaroxaban 15mg 🡪 20mg** | **Creatinine Clearance (Cockcroft Gault Equation)** | >50 ml/min | >55 ml/min |

**Figure D1. Subgroup Analysis of Scenarios Further from Cut-Off Threshold Values for Age, Weight, and/or Renal Function: Change to DOAC prescription by notification recipient in 7, 14, and 30 days**

Note: For the subgroup of notifications that were triggered due to patients’ age, weight, and/or renal function being further from cut-off threshold values, average marginal effects and 95% confidence intervals were estimated for three outcomes, the proportion of notifications resulted in changes within 7 days, 14 days, and 30 days from the notification being sent. Full model estimates for each model are shown in Appendix Table E.2

**Table D2. Full results for the post-hoc, subgroup analysis**

|  | **Subgroup: Patients farther from the cutoff threshold; Change in 7 days**  **OR [95% CI]** | **Subgroup: Patients farther from the cutoff threshold; Change in 14 days**  **OR [95% CI]** | **Subgroup: Patients farther from the cutoff threshold; Change in 30 days**  **OR [95% CI]** |
| --- | --- | --- | --- |
| Pharmacist-directed message  (vs. Prescriber-directed) | 1.17 [0.52, 2.64] | 0.99 [0.48, 2.05] | 1.32 [0.52, 3.36] |
| Prescriber role: v. primary care |  |  |  |
| Trainee | 0.98 [0.09, 10.53] | 0.70 [0.07, 7.02] | 1.95 [0.15, 24.68] |
| Specialty clinic | 1.20 [0.50, 2.87] | 0.94 [0.42, 2.08] | 0.78 [0.29, 2.14] |
| (Constant) | 0.30 [0.14, 0.66] | 0.48 [0.26, 0.90] | 0.445 [0.19, 1.05] |
| BIC | 185.4154 | 193.1645 | 203.5907 |

Note: Models estimated only with the subgroup of notifications where patients’ age, weight, and/or renal function was further from cut-off threshold values, for three outcomes, the proportion of notifications resulted in changes within 7 days, 14 days, and 30 days from the notification being sent: N (number of prescribers) = 101 and n (number of notifications) = 139.

**Appendix E. Primary and secondary analyses of the primary outcomes and the sensitivity analyses**

**Figure E1. Change to DOAC prescription by notification recipient in 7, 14, and 30 days**

Note: Average marginal effect and 95% confidence intervals estimated for three outcomes, the proportion of notifications resulted in changes within 7 days, 14 days, and 30 days from the notification being sent. Full model estimates for each model are shown in Appendix Table D1

**Table E1.** **Provider-level characteristics of prescribers with and without DOAC prescriptions that met eligibility**

|  | **Prescribers with DOAC prescriptions that met eligibility (N=183)** | **Prescribers without DOAC prescriptions that met eligibility (N=1,609)** | |
| --- | --- | --- | --- |
|  | n (%) | | n (%) |
| **Role** |  | |  |
| Resident | 12 (6.6) | | 592 (36.8) |
| Primary Care | 108 (59.0) | | 309 (19.2) |
| Specialty | 63 (34.4) | | 708 (44.0) |
| **Provider Type** |  | |  |
| Nurse Practitioner | 31(16.9) | | 172 (10.7) |
| Physician | 131 (71.6) | | 601 (37.3) |
| Physician Assistant | 9 (4.9) | | 196 (12.2) |
| Resident | 12 (6.6) | | 640 (39.8) |
| **DOAC prescriptions pre-trial** |  | |  |
| Median (IQR) | Median = 26 (IQR = 14 – 42) | | Median = 1 (IQR = 0 – 6) |

**Table E2. Full results: Change to DOAC prescription by notification recipient in 7, 14, and 30 days**

|  | **Change in 7 days**  **OR [95% CI]** | **Change in 14 days**  **OR [95% CI]** | **Change in 30 days**  **OR [95% CI]** |
| --- | --- | --- | --- |
| Pharmacist-directed message  (vs. Prescriber-directed) | 0.77 [0.44, 1.35] | 0.77 [0.42, 1.39] | 0.91 [0.52, 1.59] |
| Prescriber role: v. primary care |  |  |  |
| Trainee | 1.47 [0.39, 5.50] | 1.10 [0.27, 4.57] | 1.32 [0.35, 4.96] |
| Specialty clinic | 1.36 [0.76, 2.46] | 1.09 [0.58, 2.06] | 0.92 [0.50, 1.70] |
| (Constant) | 0.28 [0.18, 0.45] | 0.35 [0.21, 0.58] | 0.41 [0.26, 0.65] |
| Bayesian Information Criterion (BIC) | 447.0148 | 475.8127 | 499.7781 |

Note: N (number of prescribers) = 183; n (number of notifications) = 388.

**Table E3. Sensitivity Analyses: Change to DOAC prescription by notification recipient, adjusted for prescriber- and patient-level clustering**

|  | **(1)**  **Change in 7 days, adding patient-level random effect**  **OR [95% CI]** | **(2)**  **Change in 14 days, adding patient-level random effect**  **OR [95% CI]** | **(3)**  **Change in 30 days, adding patient-level random effect**  **OR [95% CI]** | **(4)**  **Change in 7 days: with one notification per patient-prescriber dyad**  **OR [95% CI]** |
| --- | --- | --- | --- | --- |
| Pharmacist-directed message  (vs. Prescriber-directed) | 0.74 [0.42, 1.30] | 0.74 [0.412, 1.32] | 0.85 [0.44, 1.63] | 0.82 [0.46, 1.44] |
| Prescriber role: v. primary care |  |  |  |  |
| Trainee | 0.69 [0.19, 2.47] | 1.07 [0.27, 4.18] | 1.36 [0.29, 6.31] | 1.68 [0.45, 6.27] |
| Specialty clinic | 0.98 [0.26, 3.67] | 1.12 [0.61, 2.07] | 0.94 [0.47, 1.89] | 1.44 [0.80, 2.59] |
| (Constant) | 0.44 [0.12, 1.54] | 0.38 [0.24, 0.62] | 0.38 [0.20, 0.74] | 0.27 [0.17, 0.44] |
| BIC | 419.2 | 448.8 | 474.9 | 400.0 |

Note: Models (1)-(3) estimated with random effects at both prescriber- and patient-levels. There were 23 patients whose medications were prescribed by multiple prescribers who were assigned to a different randomization. For these three models shown in this table, these patients were randomly selected to be used for only one of the prescribers: N (number of prescribers) = 177, k (number of patients) = 320, and n (number of notifications) = 359. Model (4) estimated with a random effect at prescriber-level only, but limited to one notification per a patient-prescriber dyad: N (number of prescribers) = 183 and n (number of notifications) = 345.

**Table E4. Time (in days) from notification to change in prescription**

|  | **Prescriber-directed message** | **Pharmacist-directed message** |
| --- | --- | --- |
| **For notifications resulted in change in 7 days (primary analysis):** | n = 45 | n = 45 |
| Median (IQR) | 2 days (0 – 3 days) | 2 days (0 – 4 days) |
| **For notifications resulted in change in 14 days (sensitivity analysis):** | n = 51 | n = 53 |
| Median (IQR) | 2 days (0 – 4 days) | 3 days (1 – 5 days) |
| **For notifications resulted in change in 30 days (sensitivity analysis):** | n = 54 | n = 63 |
| Median (IQR) | 2.5 days (0 – 6 days) | 4 days (1 – 10 days) |

**Table E5. Oral Anticoagulant Switches by Randomized Arm**

| **Randomization** | **Indication** | **Triggering med** | **New med (at 7 days)** |
| --- | --- | --- | --- |
| Prescriber message | Atrial Fibrillation | Apixaban | Non-DOAC |
|  | Atrial Fibrillation | Apixaban | Non-DOAC |
|  | Atrial Fibrillation | Rivaroxaban | Apixaban |
| Pharmacist message | Atrial Fibrillation | Apixaban | Rivaroxaban |
|  | Atrial Fibrillation | Rivaroxaban | Apixaban |
|  | Atrial Fibrillation | Rivaroxaban | Apixaban |
|  | Atrial Fibrillation | Rivaroxaban | Apixaban |

**Appendix F. Moderator Analyses**

**Table F1. Full results for moderator analyses**

|  | **Prescriber-level moderator: specialty vs. primary care providers**  **OR [90% CI]** | **Prescriber-level moderator: pre-trial DOAC prescriptions**  **OR [90% CI]** | **Patient characteristic moderator: patient age >70**  **OR [90% CI]** | **Patient characteristic moderator: impaired renal function**  **OR [90% CI]** | **Patient characteristic moderator: polypharmacy**  **OR [90% CI]** | **Prescription-level moderator: Rivaroxaban vs. Apixaban**  **OR [90% CI]** | **Prescription-level moderator: dose too low vs. too high**  **OR [90% CI]** | **Prescription-level moderator: near vs. farther from the threshold**  **OR [90% CI]** |
| --- | --- | --- | --- | --- | --- | --- | --- | --- |
| Pharmacist-directed message (vs. Prescriber-directed) | 0.80 [0.44, 1.43] | 1.62 [0.86, 3.03] | 0.33 [0.04, 2.90] | 1.53 [0.42, 5.51] | **0.31 [0.10, 0.99]** | 0.40 [0.17, 0.95] | 0.73 [0.38, 1.37] | 0.58 [0.32, 1.06] |
| Prescriber role (vs. primary care) |  |  |  |  |  |  |  |  |
| Trainee | 2.62 [0.62, 11.00] | 1.25 [0.41, 3.80] | 1.48 [0.49, 4.46] | 1.44 [0.48, 4.32] | 1.78 [0.57, 5.59] | 1.67 [0.56, 4.97] | 1.40 [0.45, 4.38] | 1.54 [0.50, 4.71] |
| Specialty clinic | 1.30 [0.65, 2.62] | 1.26 [0.80, 1.99] | 1.37 [0.83, 2.26] | 1.35 [0.83, 2.21] | 1.50 [0.89, 2.53] | 1.38 [0.85, 2.25] | 1.26 [0.76, 2.09] | 1.41 [0.85, 2.33] |
| *Pharmacist* x Trainee | 0.24 [0.02, 2.72] |  |  |  |  |  |  |  |
| *Pharmacist* x Specialty clinic prescriber | 1.10 [0.41, 2.92] |  |  |  |  |  |  |  |
| Pre-trial DOAC prescription (vs. More) |  |  |  |  |  |  |  |  |
| Fewer |  | **2.60 [1.19, 5.66]** |  |  |  |  |  |  |
| Medium |  | **2.95 [1.46, 5.97]** |  |  |  |  |  |  |
| *Pharmacist* x Fewer |  | **0.30 [0.10, 0.85]** |  |  |  |  |  |  |
| *Pharmacist* x Medium |  | **0.31 [0.11, 0.83]** |  |  |  |  |  |  |
| Age > 70 (vs. ≤ 70) |  |  | 0.78 [0.25, 2.41] |  |  |  |  |  |
| *Pharmacist* x Age > 70 |  |  | 2.40 [0.27, 21.62] |  |  |  |  |  |
| CrCl ≤ 60 (vs. >60) |  |  |  | 1.72 [0.63, 4.73] |  |  |  |  |
| *Pharmacist* x CrCl ≤ 60 |  |  |  | 0.46 [0.12, 1.78] |  |  |  |  |
| With Polypharmacy (vs. without) |  |  |  |  | **0.25 [0.11, 0.58]** |  |  |  |
| *Pharmacist* x Polypharmacy |  |  |  |  | 2.98 [0.88, 10.14] |  |  |  |
| Apixaban (vs. Rivaroxaban) |  |  |  |  |  | **0.43 [0.21, 0.85]** |  |  |
| *Pharmacist* x Apixaban |  |  |  |  |  | 2.44 [0.93, 6.39] |  |  |
| Dose too low (vs. too high) |  |  |  |  |  |  | **0.37 {0.20, 0.70]** |  |
| *Pharmacist* x Dose too low |  |  |  |  |  |  | 0.79 [0.32, 1.95] |  |
| Farther from the cutoff (vs. near) |  |  |  |  |  |  |  | 1.00 [0.52, 1.92] |
| *Pharmacist* x Farther |  |  |  |  |  |  |  | 1.93 [0.79, 4.73] |
| Constant | **0.28 [0.18, 0.43]** | **0.17 [0.10, 0.28]** | 0.36 [0.12, 1.10] | **0.18 [0.07, 0.47]** | 0.84 [0.40, 1.77] |  | **0.54 [0.32, 0.93]** | **0.28 [0.18, 0.44]** |
| n (number of notifications) | 388 | 388 | 388 | 388 | 388 | 388 | 379 | 388 |
| N (number of prescribers) | 183 | 183 | 183 | 183 | 183 | 183 | 179 | 183 |
| BIC | 457.8 | 463.4 | 458.5 | 457.9 | 451.0 | 455.0 | 433.5 | 455.8 |
